# Supplementary material for: Free log-likelihood as an unbiased metric for coherent diffraction imaging
Source: Sci Rep. 2020 Feb 14;10:2664. doi: 10.1038/s41598-020-57561-2 (PMC7021796; doi:10.1038/s41598-020-57561-2)
Supplement: Supplementary file 1 — Supplementary Information. [file 41598_2020_57561_MOESM1_ESM.pdf]

Free log-likelihood as an unbiased metric for coherent diffraction imaging

Vincent Favre-Nicolin[1,2,*], Steven Leake[1], and Yuriy Chushkin[1]

[1]ESRF, The European Synchrotron, 71 Avenue des Martyrs, 38000 Grenoble, France [2]Univ. Grenoble Alpes, Grenoble, France
[*] favre@esrf.fr

Supplementary information

Free log-likelihood curve vs free pixels island size

As indicated in the main text, the free log-likelihood is calculated by setting aside "~5% of the observed diffraction data in a 'free' set of pixels, which are grouped as islands of radius 3 pixels - to make sure that correlations between neighbouring pixels does not create a strong relationship between the working set and the 'free' set."

In the following figures we have performed the same calculations and plot as for figure 2 of the article, but by changing the island size with a radius varying from 0 (isolated pixels) to 6. The radius is indicated for each figure in the legend.

As can be seen in these figures, the free log-likelihood is not discriminating enough for the small island's radii (0 and 1), as incorrect solutions with a large support still can yield small LLK_{free} values. This is particularly true when the radius is equal to zero, as the LLK_{free} has the same tendency as the normal log-likelihood, i.e. it is decreasing with an increasing number of pixels in the object support. This confirms that using islands with a sufficient radius is necessary to yield a discriminating figure of merit, in order to obtain a sufficient

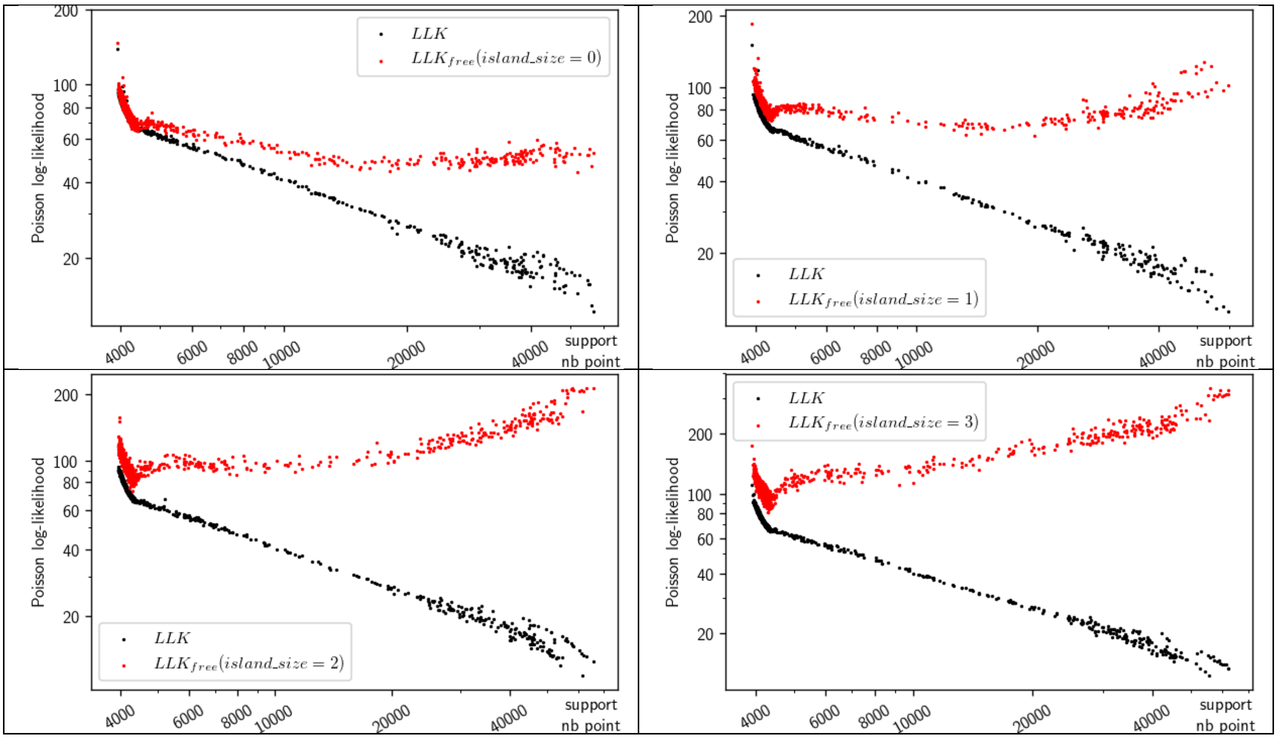

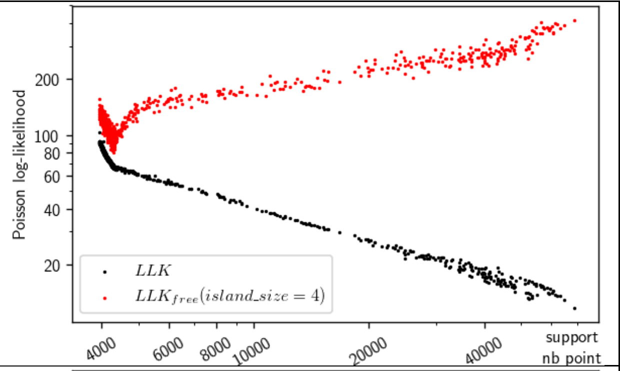

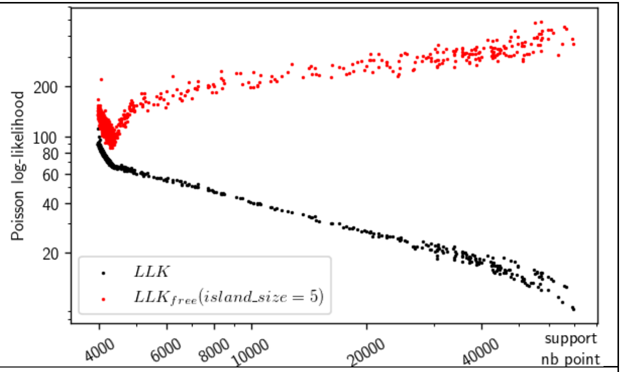

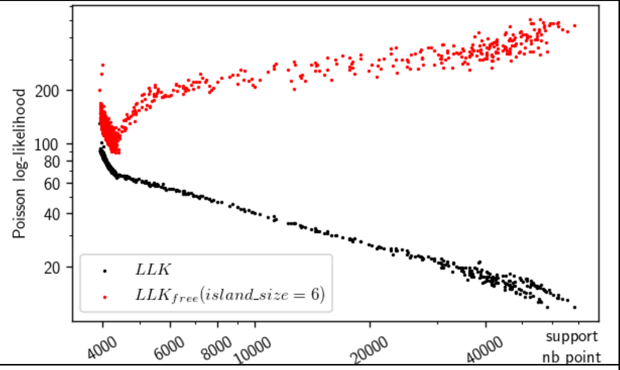

Fig. 1sup: scatter plots of log-likelihood (black) and free log-likelihood (red) vs the number of points in the support, obtained by generating 1000 solutions with random starting objects and a different threshold. Each plot corresponds to a different calculation of LLK_{free}, with 'free' pixels grouped as islands with a radius (indicated in the legend) varying from 0 to 6 pixels. Each plot also corresponds to 1000 independent solutions with different random start and a random threshold.

Free log-likelihood curve for the cyanobacteria data

This curve was generated similarly to figures 2 and 1sup, but for the cyanobacteria dataset presented in figure 4. The overall behaviour is similar to figure 2, with the normal log-likelihood decreasing with increasing number of points in the support, whereas the free log-likelihood presents a minimum around the ideal support size.

In this particular case the minimum is less pronounced than in Fig.2, due to the faceted shape of the bacteria which allows relatively easy convergence of the algorithm towards a correct shape.

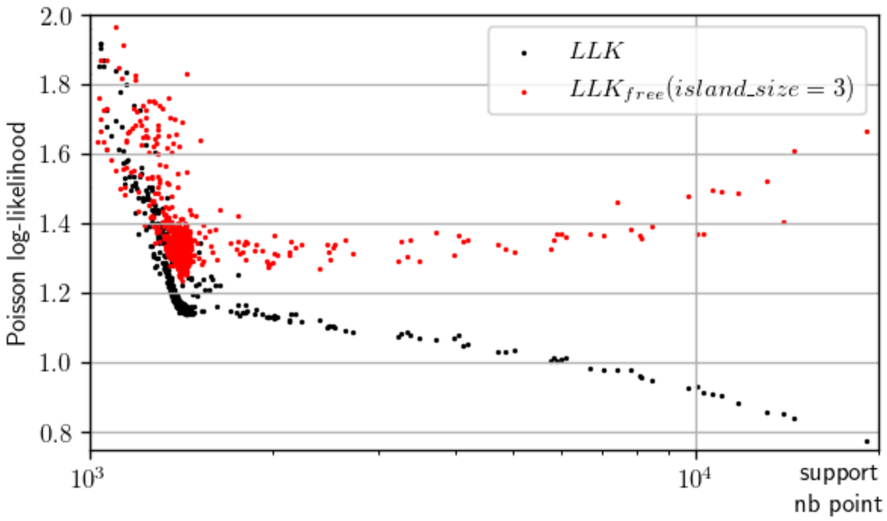

Fig. 2sup: scatter plots of log-likelihood (black) and free log-likelihood (red) vs the number of points in the support, obtained by generating 1000 solutions with random starting objects and a different threshold.
